# Notes on the Origin, Nature, Prevention, and Treatment of Asiatic Cholera

**Published:** 1867-07

**Authors:** 


Notes on the Origin, Nature, Prevention, and Treatment of Asiatic Cholera. By John C. Peters, M.D. Second Edition, with an Appendix. New York: D. VAN NOSTRAND, 192 Broadway. 1867.

This is a neatly printed duodecimo volume of 200 pages. It is written in good style; and the title-page gives a good idea of the scope of its contents.

